# Histogram analysis of tensor-valued diffusion MRI in meningiomas: Relation to consistency, histological grade and type

**DOI:** 10.1016/j.nicl.2021.102912

**Published:** 2021-12-13

**Authors:** Jan Brabec, Filip Szczepankiewicz, Finn Lennartsson, Elisabet Englund, Houman Pebdani, Johan Bengzon, Linda Knutsson, Carl-Fredrik Westin, Pia C. Sundgren, Markus Nilsson

**Affiliations:** a Medical Radiation Physics, Clinical Sciences, Lund University, Lund, Sweden; b Diagnostic Radiology, Clinical Sciences, Lund University, Lund, Sweden; c Pathology, Clinical Sciences, Lund University, Lund, Sweden; d Department of Neurosurgery, Clinical Sciences, Lund University, Lund, Sweden; e Russell H. Morgan Department of Radiology and Radiological Science, Johns Hopkins University School of Medicine, Baltimore, MD, USA; f F. M. Kirby Research Center for Functional Brain Imaging, Kennedy Krieger Institute, Baltimore, MD, USA; g Department of Radiology, Brigham and Women's Hospital, Boston, MA, USA; h Harvard Medical School, Boston, MA, USA; i Lund University Bioimaging Center, Lund University, Lund, Sweden; j Department for Imaging and Function, Skåne University Hospital, Lund University, Lund, Sweden; k Lund Stem Cell Center, Clinical Sciences, Lund University, Lund, Sweden

**Keywords:** Diffusion MRI, Tensor-valued diffusion encoding, Meningioma, Consistency, Tumor grade, Type, ADC, Apparent Diffusion Coefficient, AUC, Area Under Curve, DKI, Diffusion Kurtosis Imaging, dMRI, Diffusion Magnetic Resonance Imaging, DTI, Diffusion Tensor Imaging, DWI, Diffusion Weighted Imaging, FA, Fractional Anisotropy, FOV, Field of View, FLAIR, Fluid-Attenuated Inversion Recovery, LTE, Linear Tensor Encoding, MD, Mean Diffusivity, MK, Mean Kurtosis, MK_A_, Anisotropic kurtosis, MK_I_, Isotropic kurtosis, ROC, Receiver Operating Characteristic, ROI, Region of Interest, STD, Standard Deviation, STE, Spherical Tensor Encoding, WHO, World Health Organization

## Abstract

**Background:**

Preoperative radiological assessment of meningioma characteristics is of value for pre- and post-operative patient management, counselling, and surgical approach.

**Purpose:**

To investigate whether tensor-valued diffusion MRI can add to the preoperative prediction of meningioma consistency, grade and type.

**Materials and methods:**

30 patients with intracranial meningiomas (22 WHO grade I, 8 WHO grade II) underwent MRI prior to surgery. Diffusion MRI was performed with linear and spherical b-tensors with b-values up to 2000 s/mm^2^. The data were used to estimate mean diffusivity (MD), fractional anisotropy (FA), mean kurtosis (MK) and its components—the anisotropic and isotropic kurtoses (MK_A_ and MK_I_). Meningioma consistency was estimated for 16 patients during resection based on ultrasonic aspiration intensity, ease of resection with instrumentation or suction. Grade and type were determined by histopathological analysis. The relation between consistency, grade and type and dMRI parameters was analyzed inside the tumor (“whole-tumor”) and within brain tissue in the immediate periphery outside the tumor (“rim”) by histogram analysis.

**Results:**

Lower 10th percentiles of MK and MK_A_ in the whole-tumor were associated with firm consistency compared with pooled soft and variable consistency (n = 7 vs 9; *U* test, p = 0.02 for MK_A 10_ and p = 0.04 for MK_10_) and lower 10th percentile of MD with variable against soft and firm (n = 5 vs 11; *U* test, p = 0.02). Higher standard deviation of MK_I_ in the rim was associated with lower grade (n = 22 vs 8; *U* test, p = 0.04) and in the MK_I_ maps we observed elevated rim-like structure that could be associated with grade. Higher median MK_A_ and lower median MK_I_ distinguished psammomatous type from other pooled meningioma types (n = 5 vs 25; *U* test; p = 0.03 for MK_A 50_ and p = 0.03 and p = 0.04 for MK_I 50_).

**Conclusion:**

Parameters from tensor-valued dMRI can facilitate prediction of consistency, grade and type.

## Highlights

• Tensor-valued dMRI facilitates prediction of meningioma consistency, grade and type.
• Tensor-valued dMRI corroborates findings of diffusion tensor and kurtosis imaging.
• MK and MK_A_ is associated with firm and MD with variable meningioma consistency.
• Variability of MK_I_ in the vicinity of the tumor is associated with meningioma grade.
• MK_A 50_ and MK_I 50_ separates psammomatous meningiomas from other meningioma types.

## 1 Introduction

Meningiomas are the most prevalent primary intracranial tumors (34 %) with an annual incidence rate of approximately 8 in 100,000 (Louis et al., 2016). They are divided into 15 different histological types, among which the microstructure is highly heterogeneous (Wiemels et al., 2010). Although meningiomas are predominately benign, they often necessitate neurosurgical resection and, thus, their pre- and post-operative assessment and radiological differential diagnosis are of importance. Information on the meningioma consistency, grade and type prior to surgery would help to establish an appropriate pre- and post-operative plan and facilitate the radiological differential diagnostics. Meningioma consistency—defined as the mechanical firmness of the tumor tissue—is an important factor in the preoperative neurosurgical planning (Shiroishi et al., 2016, Yao et al., 2018). The firmness has an impact on the resection strategy and is associated with higher risk of recurrence (Keppler-Noreuil et al., 2016, Yesilöz et al., 2017, Zada et al., 2017). Preoperative assessment of firm consistency could also favor inclusion of a more experienced surgical team with longer operation time scheduled and more extensive neurophysiological monitoring. Meningioma grade affects the decision on adjuvant therapy, and higher grade meningiomas are frequently associated with microinvasion of the brain (Louis et al., 2016). Meningioma type is also relevant because some mutations are associated with particular subtypes, such as transitional and meningothelial meningiomas (Brastianos et al., 2013, Clark et al., 2013, Sahm et al., 2013). Furthermore, clear-cell subtype is associated with high recurrence rate and aggressiveness (Chen et al., 2011).

MRI is the method of choice for presurgical tumor characterization. Measures of meningioma consistency have been associated with parameters based on T2-weighted MRI (Yao et al., 2018), MR elastography (Chartrain et al., 2019) and diffusion tensor imaging (DTI) (Kashimura et al., 2007, Romani et al., 2014). Specifically, the fractional anisotropy (FA) from DTI in firm tumors was higher than in soft tumors and the mean diffusivity (MD) in firm tumors was of similar value to gray matter (Romani et al., 2014). However, not all studies found MD to be useful (Watanabe et al., 2016). Meningioma grade has been associated with tumor volume, tumor location, presence of edema on T2 FLAIR, the apparent diffusion coefficient (ADC), and the mean kurtosis (MK) from diffusion kurtosis imaging (DKI) although with limited accuracy (Gurkanlar et al., 2005, Hsu et al., 2010, Lin et al., 2018, Pistolesi et al., 2002, Santelli et al., 2010). For meningioma typing, DTI has been proposed for differentiation of atypical, fibroblastic and other benign meningiomas (Jolapara et al., 2010). In summary, no universally accepted method has yet been established for presurgical non-invasive estimation of meningioma consistency, grade and type (Yao et al., 2018).

Previous research on presurgical dMRI of meningiomas used conventional diffusion encoding (Stejskal and Tanner, 1965). This approach has a fundamental limitation, however, as it conflates microscopic diffusion anisotropy with orientation dispersion (Szczepankiewicz et al., 2016). One consequence is that low fractional anisotropy (FA) is found in both tumor tissue containing elongated cell structures that are incoherently oriented and in tumor tissue without elongated cell structures. Separation of these microstructurally different cases is not possible using methods based on conventional dMRI (Mitra, 1995), such as DTI (Basser et al., 1994) or diffusion kurtosis imaging (DKI) (Jensen et al., 2005). However, dMRI with tensor-valued encoding enables this separation by introducing a new measurement dimension—the b-tensor shape—that can be varied to obtain more information on the microstructure (Eriksson et al., 2013, Lasič et al., 2014, Westin et al., 2016). Using tensor-valued terminology, conventional encoding yields linear b-tensor encoding as it encodes for diffusion along a single direction per shot. By contrast, spherical b-tensor encoding sensitizes the signal to diffusion in all directions simultaneously (Mori and Van Zijl, 1995, Szczepankiewicz et al., 2020, Wong et al., 1995). Acquiring data with both linear and spherical encoding enables the separation of the MK from DKI into two components (Lasič et al., 2014); and using the terminology in (Szczepankiewicz et al., 2016), we refer to these as the anisotropic and isotropic kurtosis (MK_A_ and MK_I_, respectively). These have different histological correlates. High MK_A_ is in intracranial tumors associated with a high presence of elongated cell structures whereas high MK_I_ is associated with a high degree of intra-voxel variation in cell density (Szczepankiewicz et al., 2016).

The aim of this exploratory study was to test whether parameters obtained with tensor-valued dMRI can improve consistency estimation and radiological classification (grading and typing) of meningiomas, in comparison to DTI and DKI. We hypothesize that firm meningioma tumors are mainly comprised of anisotropic tissue with high microscopic diffusion anisotropy (Kashimura et al., 2007) manifesting as high MK_A_. Our second hypothesis was the measurement of MK_I_ in the surrounding brain tissue could be of value in tumor grading because low and high grade meningiomas may have different effects on the peritumoral brain tissue. Our third hypothesis was that tensor-valued dMRI is useful in meningioma typing since a previous study that reported a correlation between fibroblastic meningioma type and MK_A_ (Szczepankiewicz et al., 2016).

## 2 Materials and methods

### 2.1 Patients

This study included 30 patients with radiologically diagnosed meningioma tumors scheduled for surgical treatment between 2016 and 2018 at Skåne University Hospital, Lund, Sweden. Inclusion criteria were an age above 18 years, completed MRI examination prior to surgery, histopathologically confirmed meningioma and a signed informed consent. Furthermore, a consistency report was obtained from the neurosurgeon for 16 patients. All 30 patients were included in the analysis of type and grade and all 16 patients were included in consistency estimation.

**Fig. 1:**
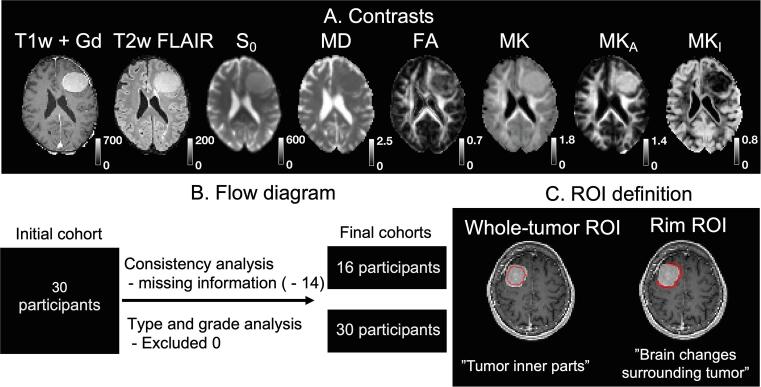
Contrast overview (panel A), flow diagram (B) and ROI definition (C). Panel A shows an example of a WHO grade I fibroblastic meningioma with variable consistency. Note that FA is high in the tumor periphery and central part but lower medially from the central part. That indicates that FA and MK_A_ reflect two different aspects - MK_A_ maps the microscopic diffusion anisotropy whereas FA shows the macroscopic diffusion anisotropy, which is lower due to low orientation coherence (Szczepankiewicz et al., 2016). Panel B shows the flow diagram of the study where the two patient populations are characterized in Table 1, respectively. The consistency analysis was performed on 16 subjects while tumor grade and tumor type analysis on 30 subjects. Panel C defines two region-of-interests (ROIs) used in the study – a “whole-tumor ROI” characterizing inner parts of the tumor and “rim ROI” characterizing the reaction in the brain tissue surrounding the tumor.

The study was approved by the Swedish Ethical Review Authority, and all subjects gave their written informed consent to participate in accordance with the Declaration of Helsinki. Table 1 provides a summary of patient demographics for the two groups and Fig. 1B flow diagram of the study.

**Table 1:** Demographics table of the patients’ for the type and grade (panels A) and consistency analysis (panels B). The patient group of the consistency analysis is the subset of patients from the type and grade analysis where the only exclusion criterion was absence of consistency report from the neurosurgeon. *All patients are classified according to WHO 2016 classification (*Louis et al., 2016*).*

		A. Type and grade analysis		B Consistency analysis	
		Frequency	Percentage	Frequency	Percentage
**Patient count**		30	100 %	16	100 %
**Age**	Mean ± standard deviation	58 ± 14 years		58 ± 15 years	
	Range (min – max)	29 – 86 years		29 – 77 years	
**Sex**	Male	13	43 %	7	43 %
	Female	17	57 %	9	57 %
**Consistency**	Firm	7	23 %	7	23 %
	Variable	5	17 %	5	17 %
	Soft	4	13 %	4	13 %
	Unknown	14	47 %	–	–
**Type**	Fibroblastic (WHO I)	6	20 %	4	25 %
	Fibroblastic (WHO II)	2	7 %	1	6 %
	Meningothelial (WHO I)	1	3 %	1	6 %
	Meningothelial (WHO II)	2	7 %	1	6 %
	Transitional (WHO I)	8	26 %	2	13 %
	Transitional (WHO II)	1	3 %	1	6 %
	Clear-cell (WHO II)	2	7 %	2	13 %
	Microcystic/Angiomatous (WHO I)	2	7 %	2	13 %
	Chordoid (WHO II)	1	3 %	0	0 %
	Psammomatous (WHO I)	5	17 %	2	13 %
**Grade**	WHO I	22	73 %	11	69 %
	WHO II	8	27 %	5	31 %
	WHO III	0	0 %	0	0 %
**Location**	Convexity	10	33 %	8	50 %
	Parasagittal	3	10 %	2	13 %
	Falx	5	17 %	1	6 %
	Sphenoid wing	5	17 %	2	13 %
	Suprasellar	1	3 %	1	6 %
	Tentorial	1	3 %	0	0 %
	Olfactory groove / Planum sphenoidale	2	7 %	0	0 %
	Clinoid / Petroclival	1	3 %	0	0 %
	Cerebellar	2	7 %	2	13 %
**Treatment**	Neurosurgery - no prior irradiation	30	100 %	16	100 %

### 2.2 Consistency quantification

All patients were treated by gross total resection of the tumor within a week after the MR examination. Perioperative evaluation of consistency during the resection was based on ultrasonic aspiration (CUSA) intensity, the ease of resection with instrumentation and suction and categorized as soft, variable, or firm.

### 2.3 Histopathologic determination of grade and type

The grade and type of the meningioma were determined by histopathological examination. Surgically removed tissue was fixed in formaldehyde solution 4 %, cut in representative sections end, embedded in paraffin and, thereafter, sectioned at 4 µm. The sections were all stained for hematoxylin-eosin, selected sections also for proliferation marker Ki-67 and glial marker GFAP to visualize grades II or III and/or brain invasion with reactive changes in adjacent brain tissue. Microstructural assessment was done according to the WHO criteria of 2016 (Louis et al., 2016).

### 2.4 MRI acquisition

MRI was performed using a 3T scanner (MAGNETOM Prisma, Siemens Healthcare, Erlangen, Germany) for pre-surgical planning within one week prior to the surgery. A prototype b-tensor encoding sequence was used to perform linear and spherical b-tensor encoding (LTE and STE, respectively) (Szczepankiewicz et al., 2019). The echo time was 80 ms, repetition time 6000 ms, in-plane acceleration factor 2 (GRAPPA), partial Fourier factor 0.75, readout bandwidth 1200 Hz/pixel, voxel size 2.3 × 2.3 × 2.3 mm^3^, FOV of 230 × 230 mm^2^ with 40 slices positioned so that the T1-weighted enhancing tumor lesion (identified in previous examinations) was in the central part of the FOV. LTE waveforms used monopolar trapezoids (Stejskal and Tanner, 1965), whereas STE was performed with numerically optimized waveforms using a maximal gradient slew rate of 50 mT/m, an energy dissipation factor of 0.5, and the max-norm constraint that inscribes the waveform within a cube that is 2 × 80 mT/m on each side (Sjölund et al., 2015). The dMRI data were acquired with b-values of 100, 700, 1400 and 2000 s/mm^2^ in 6, 6, 12, and 16 directions for LTE while for STE each b-value was repeated 6, 6, 12, and 16 times, respectively. The scan time for LTE and STE together was 8 min. We also acquired T1-weighted images before and after injection of gadolinium and T2-weighted images with resolution 1 × 1 × 1 mm^3^.

### 2.5 Parameter estimation

The dMRI data were corrected for eddy currents and motion in Elastix (Klein et al., 2009) using extrapolated references (Nilsson et al., 2015) and subsequently smoothed using a 3D Gaussian kernel with a standard deviation of 1.6 mm. To obtain maps of mean diffusivity (MD) and the two diffusional kurtosis components (MK_A_ and MK_I_) we fitted the following model to the directionally-averaged diffusion-weighted signal (S) (Nilsson et al., 2020, Westin et al., 2016):

(1) $$\log S\left(b,b_{\Delta }\right)=logS_{0}-b\cdot MD+b^{2}\cdot \left(MK_{I}+b_{\Delta }^{2}\cdot MK_{A}^{2}\right)\cdot MD^{2}/6$$

where S_0_ is the non-diffusion weighted signal, *b* is the conventional b-value, *b*_Δ_ is the shape of the b-tensor, such that *b*_Δ_ = 1 for LTE and *b*_Δ_ = 0 for STE (Eriksson et al., 2013). MK_A_ and MK_I_ were constrained to the interval between −1 and 4 to avoid outlier values. We also analyzed the data using DTI (Basser et al., 1994) and DKI (Jensen et al., 2005) to estimate the conventional FA and MK. This analysis was based on the LTE data only since it is not adapted to use of STE data. In summary, the meningiomas were characterized by the following dMRI parameters: MD, FA, MK, MK_A_, MK_I_ and for comparison S_0_.

### 2.6 ROI definition

All analysis took place in the image space of the dMRI data. To delineate the tumor, T1w and T2w images were co-registered and downsampled to match the dMRI resolution. Two types of region-of-interests (ROIs) were delineated. The first type was drawn to characterize the tumor itself. J.B. (physician) drew ROIs based on T1w + Gd images to include the maximum extent of the tumor region—here referred to as a “whole-tumor ROI.” J.B. was blinded to all dMRI maps except the S_0_ map, which was used to exclude parts of the tumor with insufficient signal, voxels with high levels of partial volume effects or those that may contain a position-dependent bias due to concomitant gradients (Szczepankiewicz et al., 2020). The second type was drawn to capture brain tissue changes in the vicinity of the tumor. J.B. drew ROIs in the brain surrounding parenchyma adjacent to the tumor-enhancing region. This ROI was up to a maximum of 2 voxels (corresponding to maximum of 4.6 mm) wide—here termed the “rim ROI.” Meninges were not included in this ROI. For each ROI, we calculated six distribution characteristics for the dMRI parameters (10th, 25th, 50th, 75th, and 90th percentile and standard deviation).

### 2.7 Statistical analysis

We performed univariate analyses of the dMRI parameter distribution characteristics to investigate whether these metrics could distinguish tumors of different consistency on a group level (for each consistency separately against pooled other two consistencies), grade (grade I vs grade II) and type (each separately against all other pooled types) using a two-sided unpaired *U* test (Wilcoxon rank sum test or Mann-Whitney test) at significance threshold of 0.05. Non-parametric tests have an inferior statistical power but higher robustness to potential outliers in non-normal distributions, which in combination with small group sizes can otherwise skew the results. The goal was to test whether we can distinguish a given consistency, grade or type from the remaining ones. To identify which of the distribution characteristics is the most useful, we analyzed the effect size of each by Cohen’s d, defined as

(2) $$d=\frac{mean\left(d_{1}\right)-mean\left(d_{2}\right)}{s}$$

where *d*_1_ and *d*_2_ are the dMRI parameter values for given distribution characteristics between the measured and pooled consistency, type or grade (e.g. mean of the 10th percentile of the MD distribution in the whole-tumor ROI of variable and firm tumors pooled subtracted from the soft ones) and *s* is pooled standard deviation defined as

(3) $$s=\sqrt{\frac{\left(n_{1}-1\right)s_{1}^{2}+\left(n_{2}-1\right)s_{2}^{2}}{n_{1}+n_{2}-2}}$$

where *n*_1_ and *n*_2_ are the number of the elements in the first and second distribution, respectively, and $s_{1}^{2}$ and $s_{2}^{2}$ are variances for these distributions. The obtained effect sizes were averaged across all dMRI parameters and all combinations of characteristics and a single number describing average effect size for given dMRI distribution. The one with the highest effects size was selected for further investigation.

In summary, the meningiomas were characterized by consistency (soft, variable, or firm), type (seven categories) and grade (grade I and II). Note that grade III and seven rare meningioma types were not present in the patient population. Each tumor was also characterized in diffusion MRI by six distribution parameters (10th, 25th, 50th, 75th, 90th percentiles and standard deviation) for each six dMRI parameters (MD, FA, MK, MK_A_, MK_I_ and for comparison S_0_) in two different ROIs (whole-tumor, rim). Finally, we also calculated area under curve (AUC) of the receiver-operating characteristics (ROC) curves and estimated the confidence intervals based on bootstrapping (n = 5000).

### 2.8 Data accessibility

The data were processed by a software package for diffusion MRI available at https://github.com/markus-nilsson/md-dmri (Nilsson et al., 2018). Analysis code, MRI protocol, and diffusion encoding gradient waveforms are available at https://github.com/jan-brabec/tensor_valued_meningiomas_in_vivo. Other data are available from the corresponding author upon request.

## 3 Results

Image contrasts used in this study included two obtained from morphological imaging (T1w + Gd and T2w FLAIR; downsampled and co-registered to the dMRI space), three from DTI (S_0_, MD and FA), one from DKI (MK), and two uniquely obtained by to tensor-valued dMRI – anisotropic and isotropic kurtoses (MK_A_ and MK_I_, respectively). The maps are displayed in Fig. 1A and show that FA and MK_A_ yields complementary information: FA shows high values only in the periphery and the core of the tumor whereas MK_A_ is relatively homogeneous but with lower value towards the tumor periphery. An overview of all cases can be found in the supplementary material. For consistency estimation, we included 16 patients and for grading and typing 30 patients (see Fig. 1B for a study flow diagram and patient characteristics for the two groups are found in Table 1). Two region-of-interests were used to obtain dMRI parameters—whole-tumor ROI and rim ROI (Fig. 1C).

**Fig. 2:**
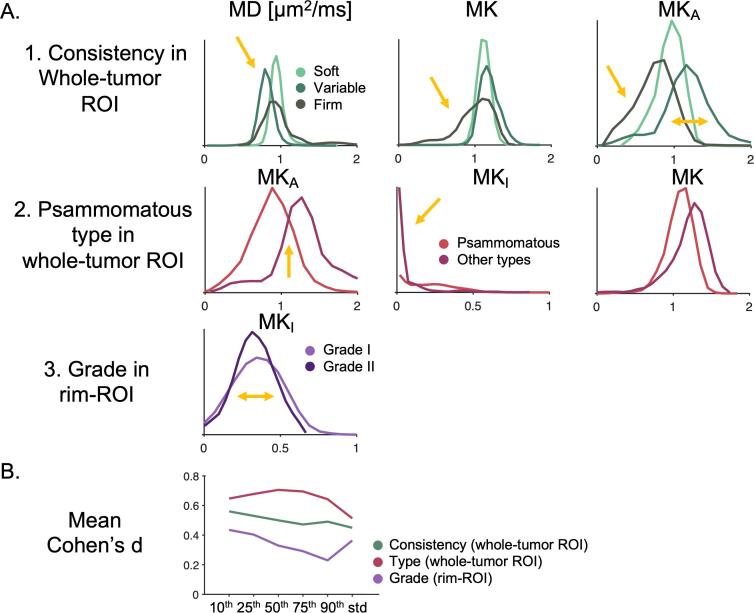
Histograms of dMRI parameters. Panel A shows distributions of parameter-values within the ROIs. Part 1 shows MD, MK and MK_A_ distributions in the whole-tumor ROI among soft, variable, and firm consistency (distribution differences indicated by yellow arrows). Part 2 shows distributions of MK_A_, MK_I_ and MK in psammomatous and other meningioma types in the whole-tumor ROI. Part 3 shows the MK_I_ distribution in grade I and II meningiomas within the rim ROI. The histograms suggest that it may be valuable to consider different distribution characteristics—their percentiles or standard deviation—shown by yellow arrows. Panel B shows effect sizes for different distribution characteristics (10th, 25th, 50th, 75th, 90th and standard deviation) as measured by Cohen’s d (defined according to Eq. (2)) averaged across all dMRI parameters (MD, FA, MK, MK_A_ and MK_I_ and S_0_). For consistency, grade and type the highest effect size is found for the 10th, 10th, and 50th percentile, respectively. For the case of grade, however, only standard deviation within MK_I_ was significantly different between the grades. (For interpretation of the references to colour in this figure legend, the reader is referred to the web version of this article.)

Histogram analysis shows which distribution characteristics that best discriminated tumors based on consistency, grade, and type (Fig. 2A). Tumors of variable consistency had its MD distribution shifted towards lower values and displayed a wider MK_A_ distribution. Tumors of firm consistency had a lower tail in MK. MK_A_ and MK_I_ separated the psammomatous type from the rest and the distribution width of MK_I_ in the rim showed a slight difference between low- and high-grade tumors. Quantitatively, based on mean Cohen’s d (defined according to Eq. (2)), the 10th percentile gave the highest effect size for the consistency, while the 50th percentile gave the highest effect size for type. Although the 10th percentile gave the highest effect size for grade the only statistically significant parameter was based on the standard deviation and, here, we selected the standard deviation distribution. That is why we have further considered for further analyses only these distributions, although other distributions had also associated some statistically significant parameters (complete overview in the supplementary material).

**Fig. 3:**
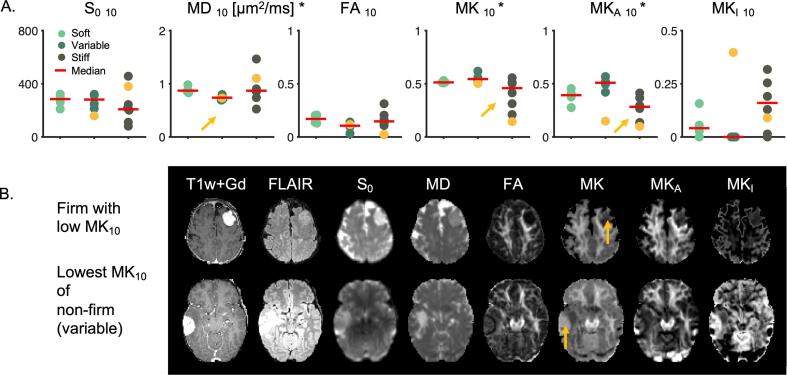
Consistency estimation. Panel A shows dMRI parameters (10th percentile within whole-tumor ROI) versus meningioma consistency. In total we included 16 patients (with meningiomas of 7 firm, 5 variable and 4 soft). Based on MK_A 10_ and MK_10_ the firm consistency is significantly different from pooled soft and variable consistency (*U* test, p = 0.04 for MK_10_, p = 0.02 for MK_A 10_). Based on MD_10_, the variable consistency can be distinguished from soft and firm one (*U* test, p = 0.02). Significant parameters are marked with an asterisk (*), significant distributions with yellow arrows. Panel B shows two examples from panel A where MK_10_ may be useful on the individual level (marked by yellow markers in panel A). The top row shows a firm tumor that is considerably darker on the MK map in comparison to the non-firm tumor in the bottom row (yellow arrows). The images are scaled according to scale bars from Fig. 1. (For interpretation of the references to colour in this figure legend, the reader is referred to the web version of this article.)

Results concerning consistency utilized the 10th percentile characteristic of the whole-tumor ROI and are shown in Fig. 3. MK_10_ and MK_A 10_ were significantly different in meningiomas of firm and pooled soft and variable consistency (n = 7 against n = 9; two-sided *U* test; p = 0.04 for MK_10_ and p = 0.02 for MK_A 10_). MD_10_ was significantly different in tumors of variable consistency compared with those of soft and firm consistency pooled (n = 5 against n = 11, two-sided *U* test; p = 0.02). An example of how these differences manifest in individual cases is shown in Fig. 3B. The upper row shows a firm meningioma with low MK_10_ whereas the bottom row a non-firm (variable) meningioma with the lowest MK_10_. Stiff meningiomas are of somewhat lower intensity in the MK map. If MK_10_ alone was used to discriminate firm consistency with a threshold 1.0 it would in this cohort yield specificity and sensitivity of 100 % and 75 %, respectively. We also note that the standard deviation of MK_A_ from Fig. 2A in panel 1 distinguished the variable consistency (p = 0.04; two-sided *U* test).

**Fig. 4:**
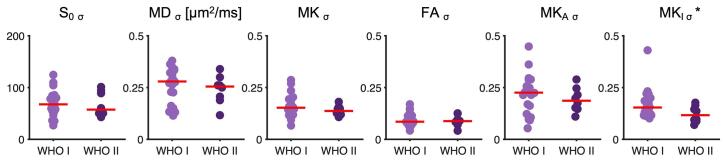
Grade estimation. Panel A shows a grade I versus grade II comparison of the standard deviation in the rim ROI for the different dMRI parameters. Standard deviation within the rim ROI of MK_I_ of grade I was significantly higher than that of grade II meningioma (n = 22 vs 8; *U* test; p = 0.04). All tumors were classified according to WHO 2016 classification (Louis et al., 2016).

**Fig. 5:**
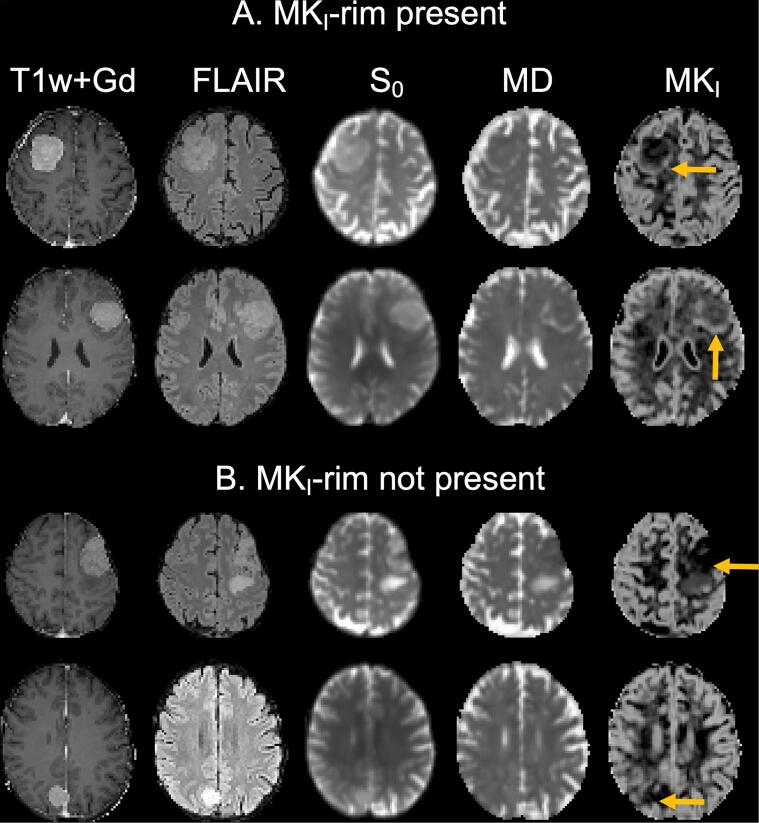
MK_I_-rim as a radiological feature. We have observed a presence of elevated MK_I_ values in a rim-like structure that partially circumscribes the tumor (panel A) or its absence (panel B). Yellow arrows mark the MK_I_-rim in panel A or tumor region in panel B, respectively. The MK_I_-rim is found in the brain tissue surrounding the T1w + Gd enhancing tumor lesion and it was preferentially present in high grade meningiomas. All cases can be found in the supplementary material. (For interpretation of the references to colour in this figure legend, the reader is referred to the web version of this article.)

Concerning grade, the only parameter that showed a significant difference between low and high grade meningiomas was the standard deviation of MK_I_ within the rim (n = 22 against n = 8; two-sided *U* test, p = 0.04) (Fig. 4). This result was corroborated by a visual finding that we refer to as an “MK_I_-rim”. This sign was preferentially present in high grade tumors and can be described as the presence of elevated MK_I_ in a rim-like structure that partially circumscribes the tumor. Note that this rim is found in the brain tissue surrounding the T1w + Gd enhancing tumor lesion. Examples are show in Fig. 5, but all cases can be found in the supplementary material.

**Fig. 6:**
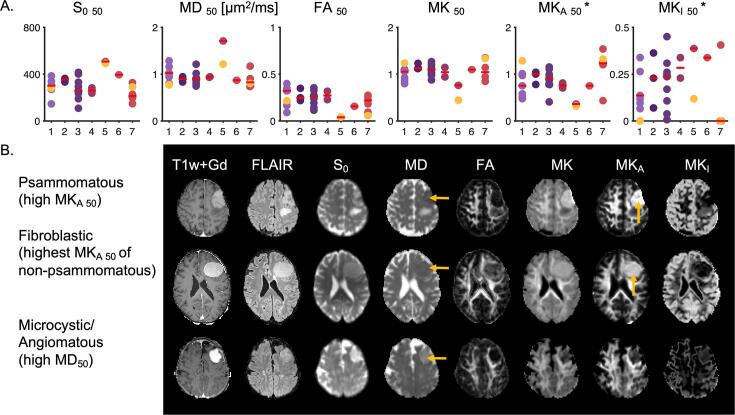
Type estimation. Panel A shows 50th percentile (median) of dMRI parameters versus meningioma type, where 1 = Fibroblastic, 2 = Meningothelial, 3 = Transitional, 4 = Clear-cell, 5 = Microcystic/Angiomatous, 6 = Chordoid, 7 = Psammomatous. The microcystic/angiomatous type (#5) is significantly different from the other types in S_0_, MD, FA, MK, MK_A_, however, only two cases were included. The psammomatous type (#7) is significantly different from the others in MK_A 50_ and MK_I 50_ but not in MK_50_ (n = 5 vs 24; *U* test; p = 0.03 for MK_A 50_ and p = 0.03 and p = 0.04 for MK_I 50_). Panel B shows three examples from panel A (marked by yellow markers). The one in the top row shows a psammomatous type with high MK_A 50_. The middle row shows a case of non-psammomatous (fibroblastic) type with the highest MK_A 50_. The psammomatous type is somewhat brighter in MK_A_ map than the highest non-psammomatous type (yellow arrows). The bottom row shows that an example of MD being generally high in a microcystic/angiomatous meningioma (yellow arrows). The images are scaled according to scale bars from Fig. 1. All tumors were classified according to WHO 2016 classification (Louis et al., 2016). (For interpretation of the references to colour in this figure legend, the reader is referred to the web version of this article.)

For classification of meningiomas into fibroblastic, meningothelial, transitional, clear-cell, microcystic/angiomatous, chordoid, and psammomatous types, the median of the parameters within the ROI had the highest effect size and this feature is shown in Fig. 6A. The psammomatous subtype was significantly different from all other types pooled (n = 5 vs n = 25) based on MK_A 50_ (p = 0.03) and MK_I 50_ (p = 0.04) but not MK_50_ (p = 0.37). Furthermore, the microcystic/angiomatous type from all other types pooled (n = 2 vs n = 28) was significantly different from the rest in S_0 50_ (p = 0.02), MD_50_ (p = 0.03), FA_50_ (p = 0.02), MK_50_ (p = 0.02) and MK_A 50_ (p = 0.02). We also note that microcystic/angiomatous type of grade I is significantly different from other types associated with grade II meningiomas (p = 0.04). However, the results are inconclusive due to the small sample size for this type (n = 2 versus n = 8) but in line with other studies (Jolapara et al., 2010, Xiaoai et al., 2020). The characteristics of psammomatous and microcystic-angiomatous meningiomas may be observed visually in the individual cases, however, as shown in the panel B. The top row shows a case with a tumor of the psammomatous type and the middle row a non-psammomatous meningioma with the highest MK_A 50_. The psammomatous type is brighter on the MK_A_ map. The bottom row shows a case of microcystic/angiomatous type that has higher MD_50_. We further note that consistency was not significantly correlated with tumor type (Table 4 and Fig. 17 in supplementary material).

**Fig. 7:**
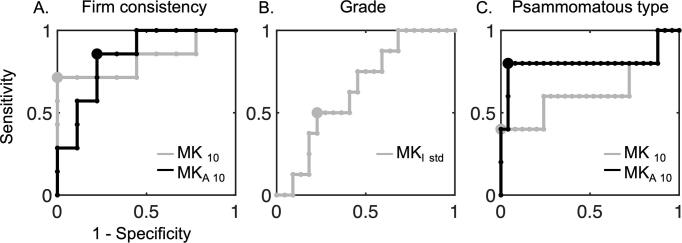
Receiver-operating characteristic (ROC) curves for the 3 cases from Fig. 1. Panel A shows ROC curves for predicting firm consistency (n = 7) from the pooled soft and variable (n = 9) based on MK_10_ (gray line) and MK_A 10_ (black line) in the whole tumor-ROI. Based on MK_10_ the AUC is 0.83 with confidence interval [0.43; 1.00] with optimal cut-point value (gray dot) with specificity 100 % and sensitivity 71 %. Based on MK_A 10_ the AUC is 0.84 with confidence interval [0.52; 0.98] with optimal cut-point value (black dot) with specificity 78 % and sensitivity 86 %. Panel B shows ROC curve for predicting grade II (n = 8) from grade I (n = 22) based on MK_I std_ in the rim-ROI. The AUC is 0.65 with confidence intervals [0.42; 0.84]. Optimal cut-point value (gray dot) yields specificity of 77 % and sensitivity 50 %. Finally, panel C shows ROC curve for prediction of psammomatous type based on MK_50_ and MK_A 50_. The AUC for MK_50_ is 0.63 with confidence interval [0.18; 1.00] with optimal cut-point value with specificity of 100 % and sensitivity 40 % (gray dot) and for MK_A 50_ 0.81 with confidence intervals [0.07; 1.00] with optimal cut-point value with specificity 96 % and sensitivity 80 %. Large confidence intervals (not shown graphically in the figure) in all cases highlight limitations of the small dataset and thus limit the interpretability of the AUC value.

Fig. 7 shows ROC curves for three of these significant tests. Distinguishing firm consistency from the pooled soft and variable based on MK_10_ and MK_A 10_ in the whole tumor-ROI yields an AUC of 0.83 with 95% confidence interval of [0.43; 1.00] and 0.84 with 95% confidence interval [0.52; 0.98], respectively (panel A). The optimal cut-point value yields for the MK_10_ a specificity of 100 % and a sensitivity of 71 %. For MK_A 10_, corresponding numbers were 78 % and 86 %, respectively. Furthermore, predicting grade II from grade I based on MK_I std_ in the rim-ROI yields an AUC of 0.65 with 95% confidence intervals of [0.42; 0.84] (panel B). The optimal cut-point value yields a specificity of 77 % and a sensitivity of 50 %. Finally, prediction of psammomatous type based on MK_50_ and MK_A 50_ has an AUC of 0.63 with a 95% confidence interval of [0.18; 1.00] and 0.81 with confidence interval [0.07; 1.00], respectively. The optimal cut-point values have specificity of 100 %, sensitivity 40 % and specificity 96 % and sensitivity 80 %, respectively.

## 4 Discussion

This pilot study aimed to explore the use of tensor-valued dMRI for presurgical characterization of meningioma tumors. Our results suggest that it may add to the characterization of tumor consistency, grade, and type—although it should be noted that the sample size was limited. Importantly, our study demonstrated examples where parameters from tensor-valued dMRI, but not from DTI nor DKI, were sensitive meningioma type and grade (Tables 1–3 in supplementary material). For example, MK_A,50_ and MK_I,50_ but not its sum that is assessed in DKI—MK_50_—can distinguish the psammomatous type of meningioma. Furthermore, the only parameter that was different between grades was MK_I std_. Tensor-valued dMRI could thus be of interest in future studies of meningiomas, in particular since high-quality results could be obtained even in just 3-minutes using a parsimonious sampling scheme and reduced field of view (Nilsson et al., 2020).

Tensor-valued dMRI is different from conventional DTI because it yields an index of the microscopic diffusion anisotropy (MK_A_), whereas FA from DTI yield an index of the voxel-level macroscopic diffusion anisotropy. In other words, FA conflates microscopic anisotropy with orientation dispersion (Szczepankiewicz et al., 2015). This may explain why MK_A_ could distinguish firm tumors, but FA could not (Fig. 3A). An underlying reason may be that the tumor consistency (based on ultrasonic aspiration intensity, ease of resection with instrumentation and suction) is related to the presence of elongated (i.e. anisotropic) cellular structures in the tissue. For example, soft tumors had a higher standard deviation of FA but lower standard deviation of MK_A_ (Table 1 in supplementary material). This may suggest that the soft meningiomas contain environments with a mixture of orientations, which could also explain the different appearance of FA and MK_A_ (Fig. 1A). Some studies have found that higher FA values are associated with firm consistency (Kashimura et al., 2007, Romani et al., 2014, Tropine et al., 2007) but our study as well as others did not find such an association (Ortega-Porcayo et al., 2015). Furthermore, we found that lower MD was associated with variable consistency. This is in line with (Miyoshi et al., 2020, Romani et al., 2014, Yogi et al., 2014) who also found MD to be useful in consistency prediction but other studies did not reproduce this result (Watanabe et al., 2016). Future *meta*-analyses may clear the picture. To facilitate this, all parameter values are reported in the supplementary material (Tables 5–7).

An important finding was that the standard deviation of MK_I_ in the near vicinity of the tumor may be associated with meningioma grade (Fig. 4). The biological underpinnings of this finding remain elusive, however. Since it is outside of the Gd-enhancing lesion, it may be gray matter that is variably compressed. It may also be related to the presence of peritumoral edema (Hale et al., 2018) or possibly indicate microinvasions, which are characteristic for higher grade meningiomas (Bi et al., 2018). The first and last interpretations are corroborated by our observation of a radiological sign that we refer to as the presence of an “MK_I_-rim”, with elevated MK_I_ around the tumor, in the high grade meningiomas. Further studies with larger cohorts are, however, needed to evaluate this observation.

Radiomics analysis is another a promising approach for meningiomas grading, typing (Park et al., 2019, Zhu et al., 2019) and consistency estimation (Zhai et al., 2021). Radiomic features for meningioma characterization has utilized contrasts of T2-weighted, T1-weighted post-contrast, and ADC maps (Cepeda et al., 2021) which is in line with previous reports of using T2-weighted MRI as a useful imaging modality for prediction of meningioma consistency (Yao et al., 2018). Analysis by radiomics could perhaps enhance the power of DWI or ADC maps to predict meningioma grade beyond what is possible simple quantitative ROI-based analyses (Santelli et al., 2010). Meningioma consistency has also been estimated by MR elastography (Chartrain et al., 2019). Currently, radiographic features such as tumor location and volume or presence of adjacent edema on conventional T2-weighted and FLAIR (Fluid-attenuated inversion recovery) images, or tumor necrosis are considered useful predictor of meningioma grade (Hale et al., 2018). However, until now there is no widely used method clinically for preoperative classification or consistency estimation.

We identified six main limitations of this study. First, the study is exploratory. Multiple uncorrected hypothesis tests were performed, and the findings need to be validated in future studies. In total we performed around 400 tests (the biology of tumors characterized by 11 options and dMRI by 36), although not all these tests were independent. We did not correct for multiple comparisons, however, as we for this exploratory study considered false negative findings to be more problematic than false positive ones. Second, the sample size was limited. We estimate that future studies aiming at a statistical power of 0.8 need more than 15 patients in each of the groups for consistency estimation and 70 patients in total for grade estimation, before accounting for patient rejection. Third, to facilitate inter-institutional comparisons of consistency, it should be estimated by a validated objective method. Such a metric was suggested after the initiation of this study (Itamura et al., 2018). Fourth, meningiomas were classified according to WHO 2016 classification (Louis et al., 2016) because all the patients were scheduled for surgical treatment between years 2016 and 2018. However, the WHO 2021 classification has now been published (Louis et al., 2021) and our findings may not be fully applicable in this new classification. Fifth, the gradient waveforms used to tensor-valued encoding were not compensated for concomitant gradient effects (Szczepankiewicz et al., 2020), as such waveforms were not available when the study was initiated. Concomitant gradients may introduce a position-dependent bias. A visual inspection showed that some regions of the brain were indeed affected, however, these regions were excluded from the analysis to minimize the potential impact. Future studies should use waveforms with minimized concomitant gradient effects (Szczepankiewicz et al., 2020). Sixth, our analysis assumes that time-dependent effects of tensor-valued dMRI are negligible. Future studies should investigate diffusion time-dependence because if this was violated, we would expect a positive bias in MK_A_ (Lundell et al., 2019).

## 5 Conclusion

Tensor-valued dMRI corroborates findings of diffusion tensor and kurtosis imaging (DTI and DKI) in preoperative analysis of meningiomas and may facilitate consistency estimation, grading and typing.

### CRediT authorship contribution statement

**Jan Brabec:** Conceptualization, Methodology, Formal analysis, Writing – original draft, Investigation. **Filip Szczepankiewicz:** Conceptualization, Software, Writing – original draft, Writing – review & editing. **Finn Lennartsson:** Validation, Visualization, Writing – review & editing. **Elisabet Englund:** Data curation, Resources. **Houman Pebdani:** Data curation, Resources, Writing – review & editing. **Johan Bengzon:** Conceptualization, Project administration, Funding acquisition, Resources, Writing – review & editing. **Linda Knutsson:** Funding acquisition, Writing – review & editing. **Carl-Fredrik Westin:** Funding acquisition, Writing – review & editing. **Pia C. Sundgren:** Conceptualization, Supervision, Project administration, Resources, Writing – review & editing. **Markus Nilsson:** Conceptualization, Methodology, Software, Project administration, Supervision, Writing – review & editing.

## Declaration of Competing Interest

The authors declare the following financial interests/personal relationships which may be considered as potential competing interests: M.N. declares ownership interests in Random Walk Imaging, and patent applications in Sweden (1250453-6 and 1250452-8), in the USA (61/642 594 and 61/642 589), and via the Patent Cooperation Treaty (SE2013/050492 and SE2013/050493). M.N. and F.S. are inventors on pending patents pertaining to the methods presented herein. None of the other authors have any conflict of interest to disclose. We confirm that we have read the journal's position on issues involved in ethical publication and affirm that this report is consistent with those guidelines.
