# Supplementary material for: Histogram analysis of tensor-valued diffusion MRI in meningiomas: Relation to consistency, histological grade and type
Source: Neuroimage Clin. 2021 Dec 13;33:102912. doi: 10.1016/j.nicl.2021.102912 (PMC8688887; doi:10.1016/j.nicl.2021.102912)

# Supplementary material

# Overview of all cases


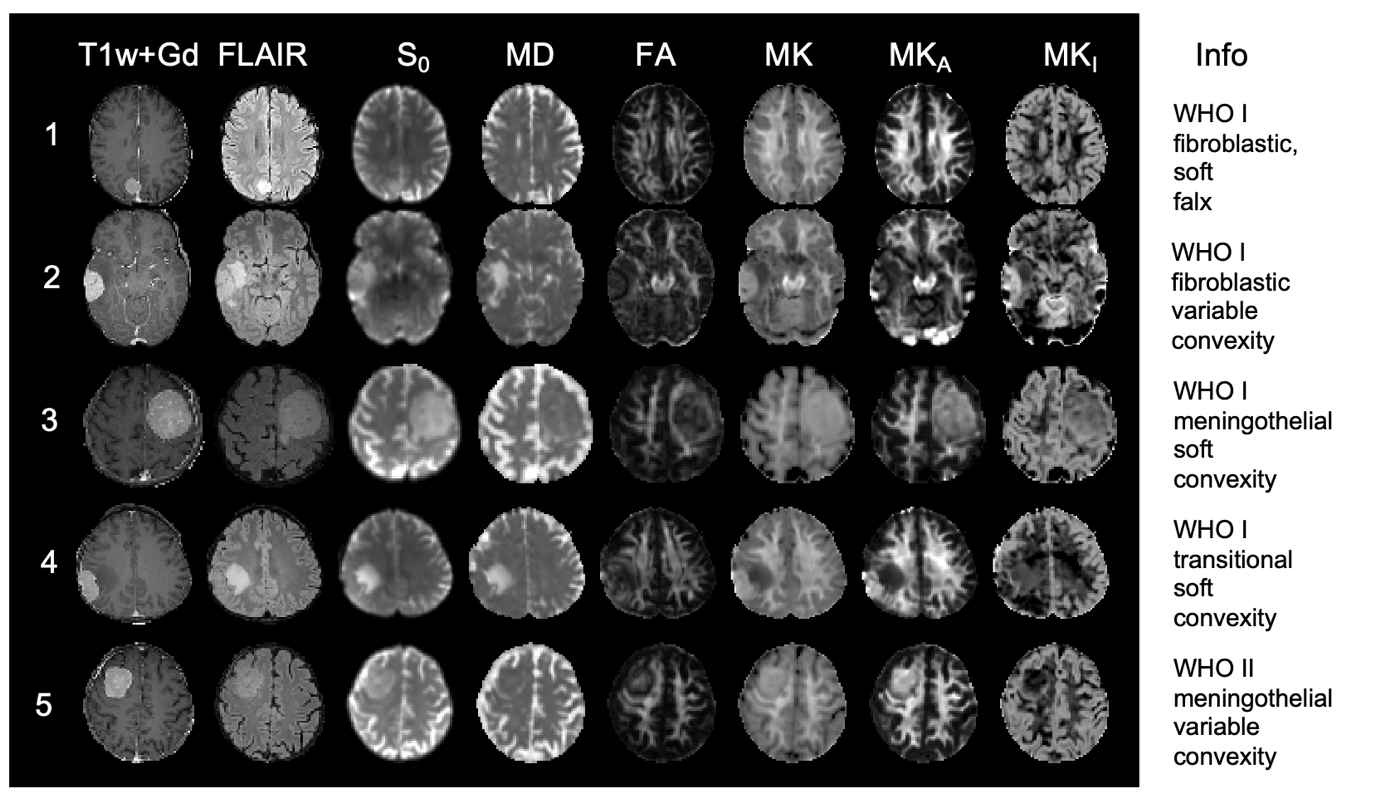


Figure 8. Complete overview of all the cases, part 1. In the rightmost column information on the grade, type, consistency and location is shown.


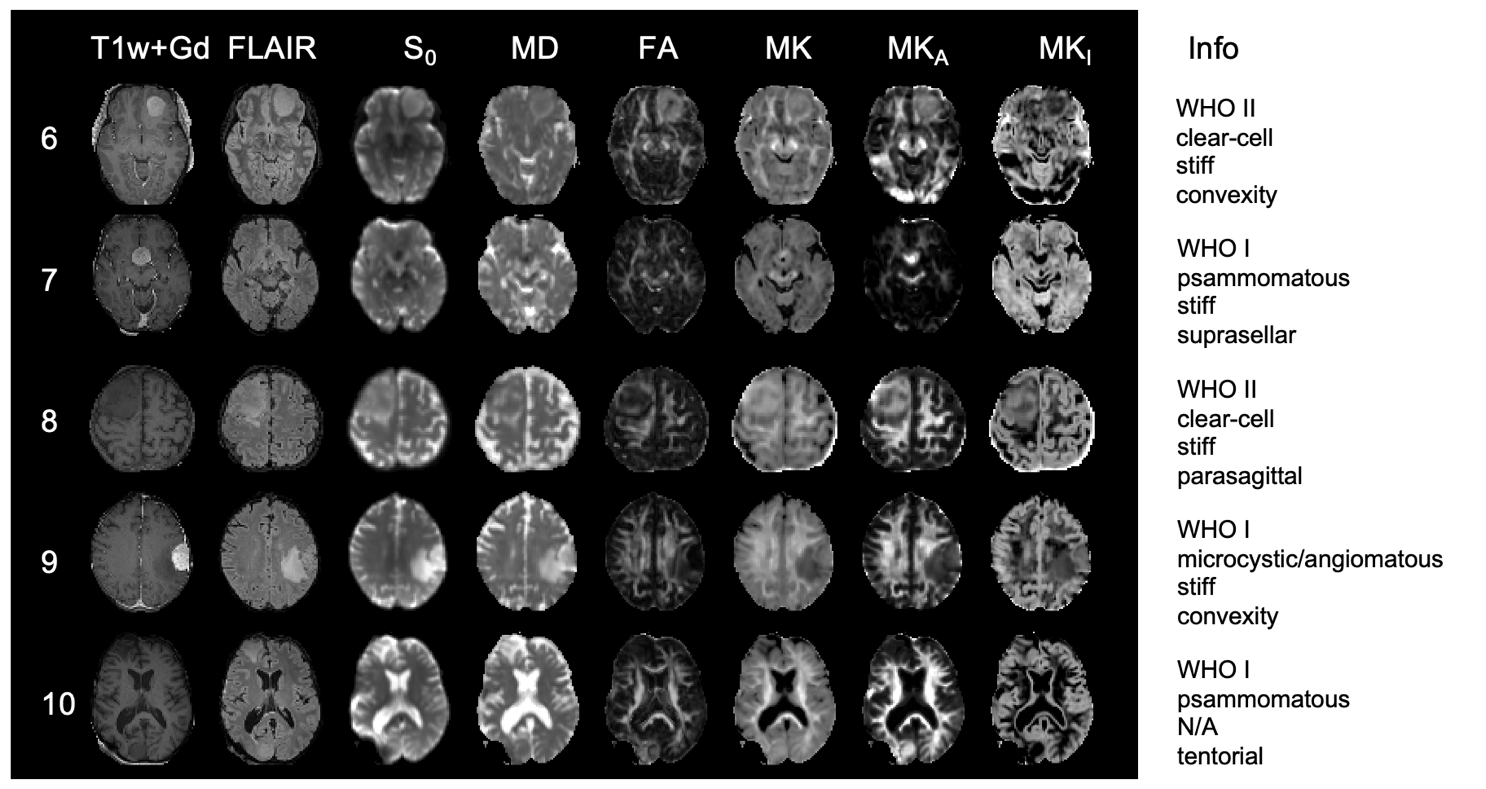


Figure 9. Complete overview of all the cases, part 2. Case 8 and 10 lack post-Gd T1w scan. In the rightmost column information on the grade, type, consistency and location is shown.


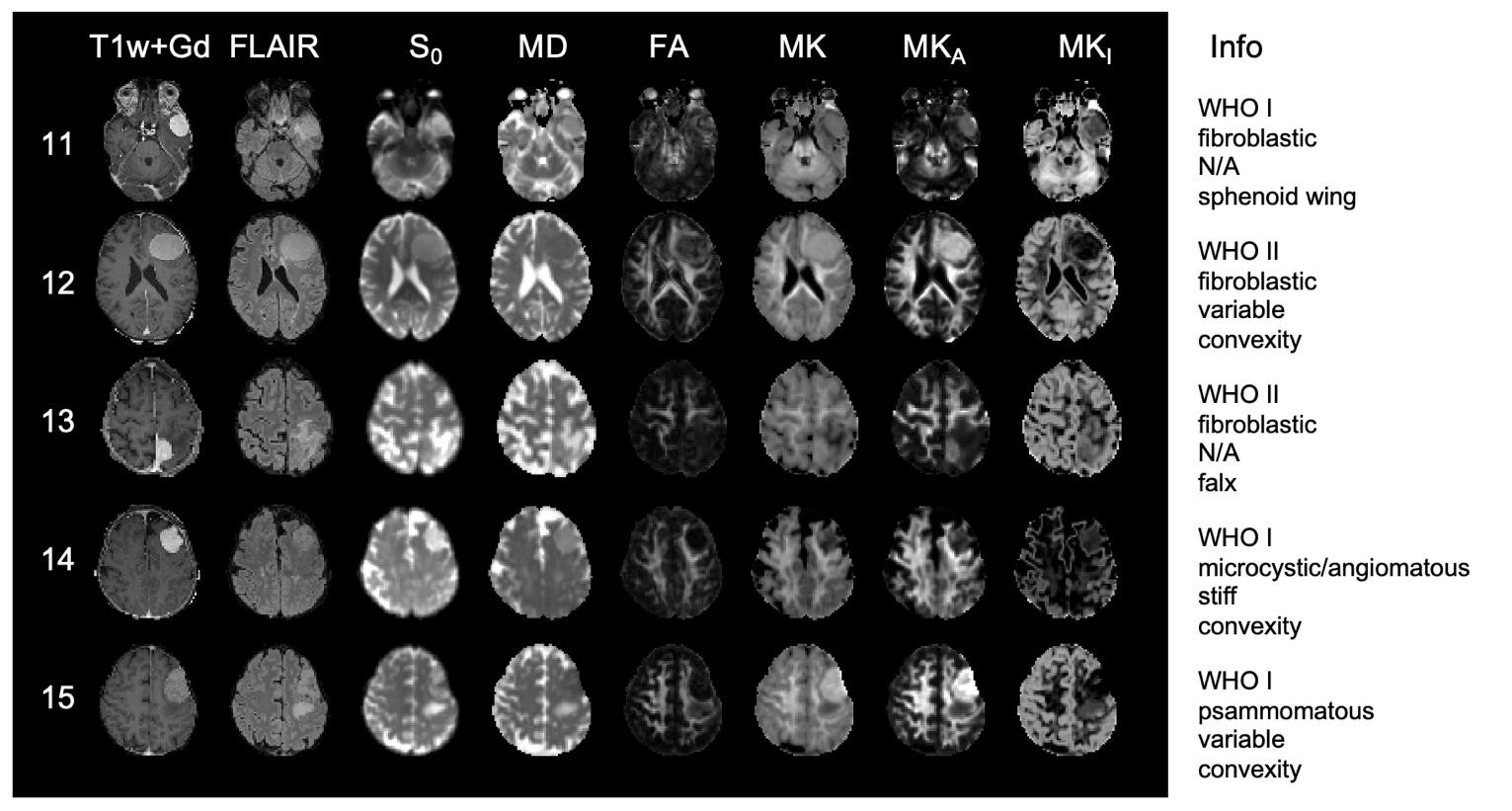


Figure 10. Complete overview of all the cases, part 3. In the rightmost column information on the grade, type, consistency and location is shown.


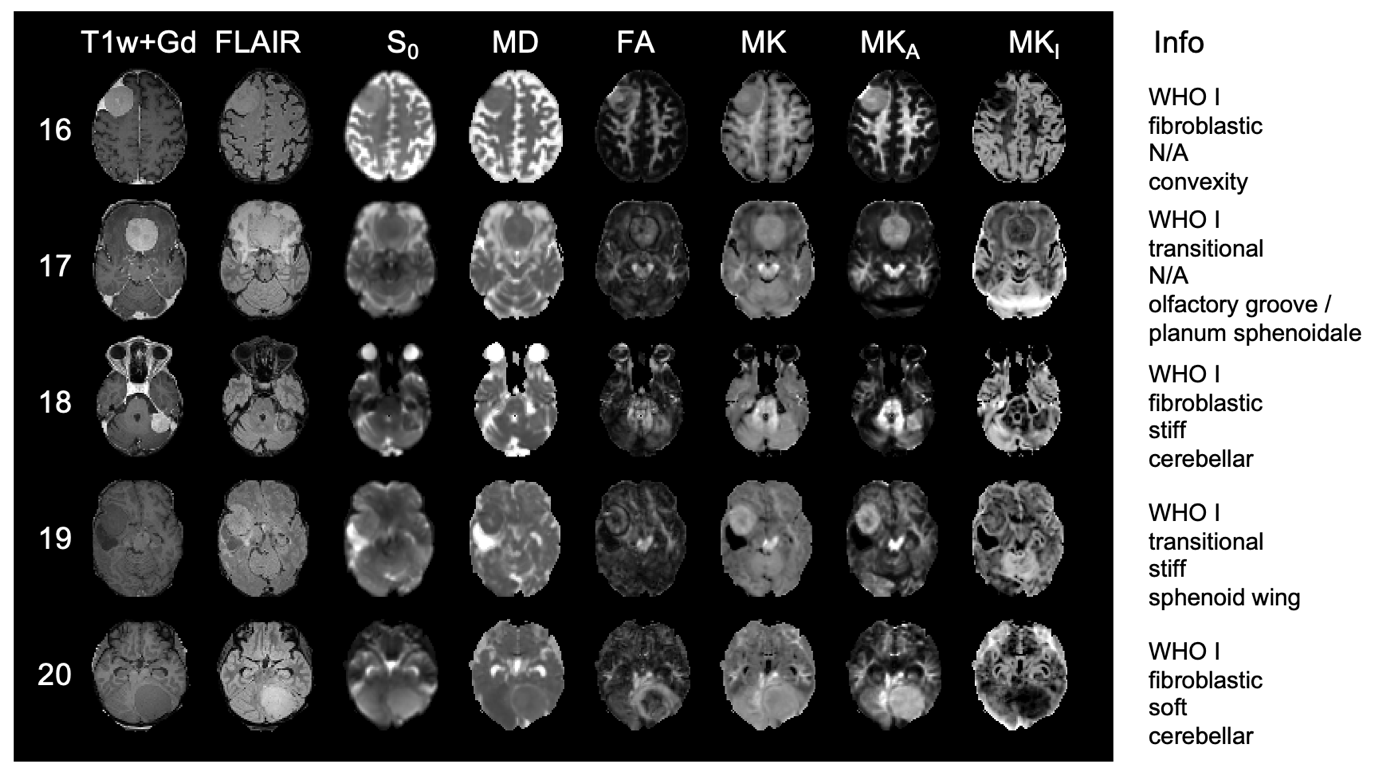


Figure 11. Complete overview of all the cases, part 4. Case 19 and 20 lack a post-Gd T1w scan. In the rightmost column information on the grade, type, consistency and location is shown.


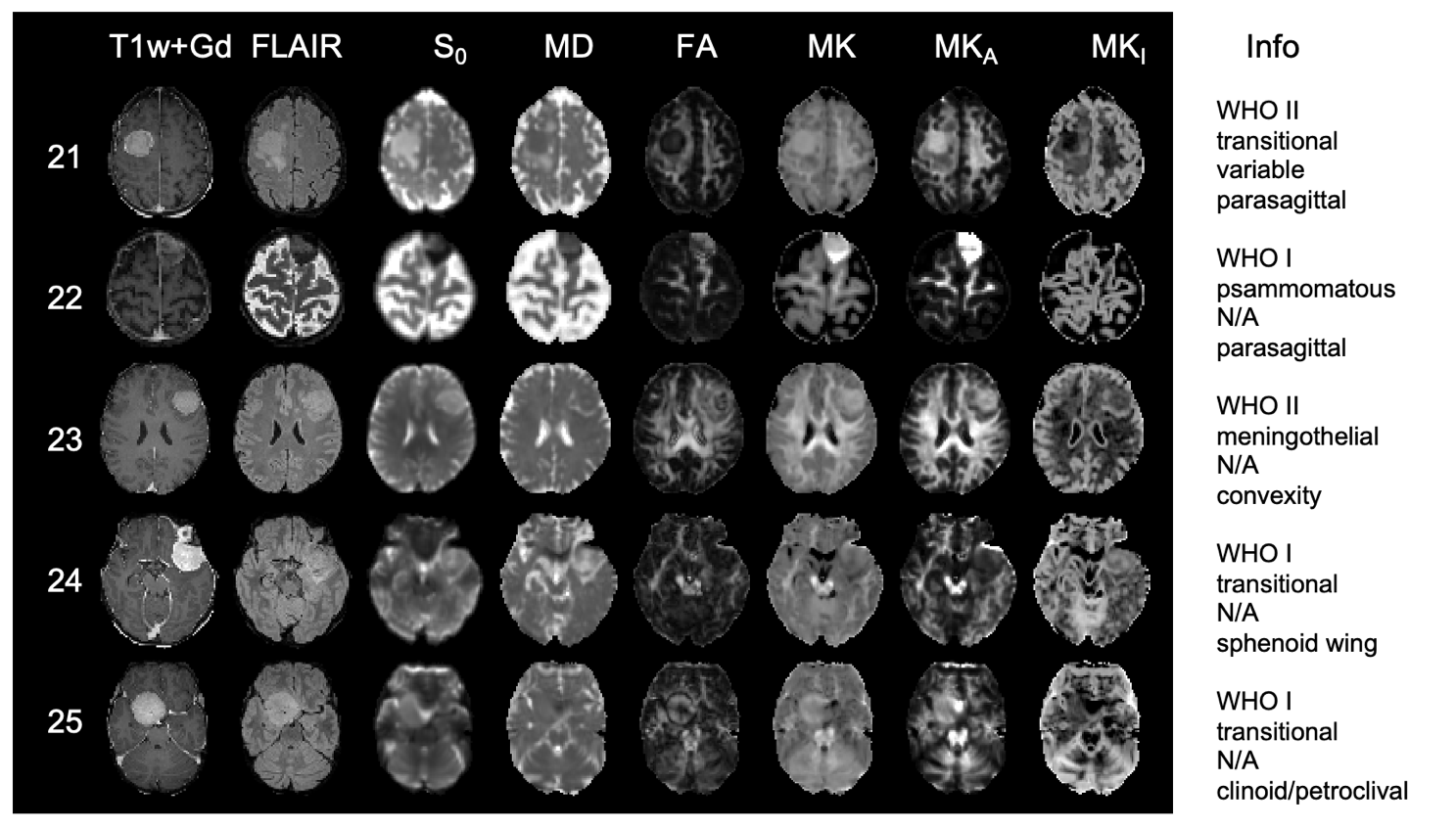


Figure 12. Complete overview of all the cases, part 5. In the rightmost column information on the grade, type, consistency and location is shown.


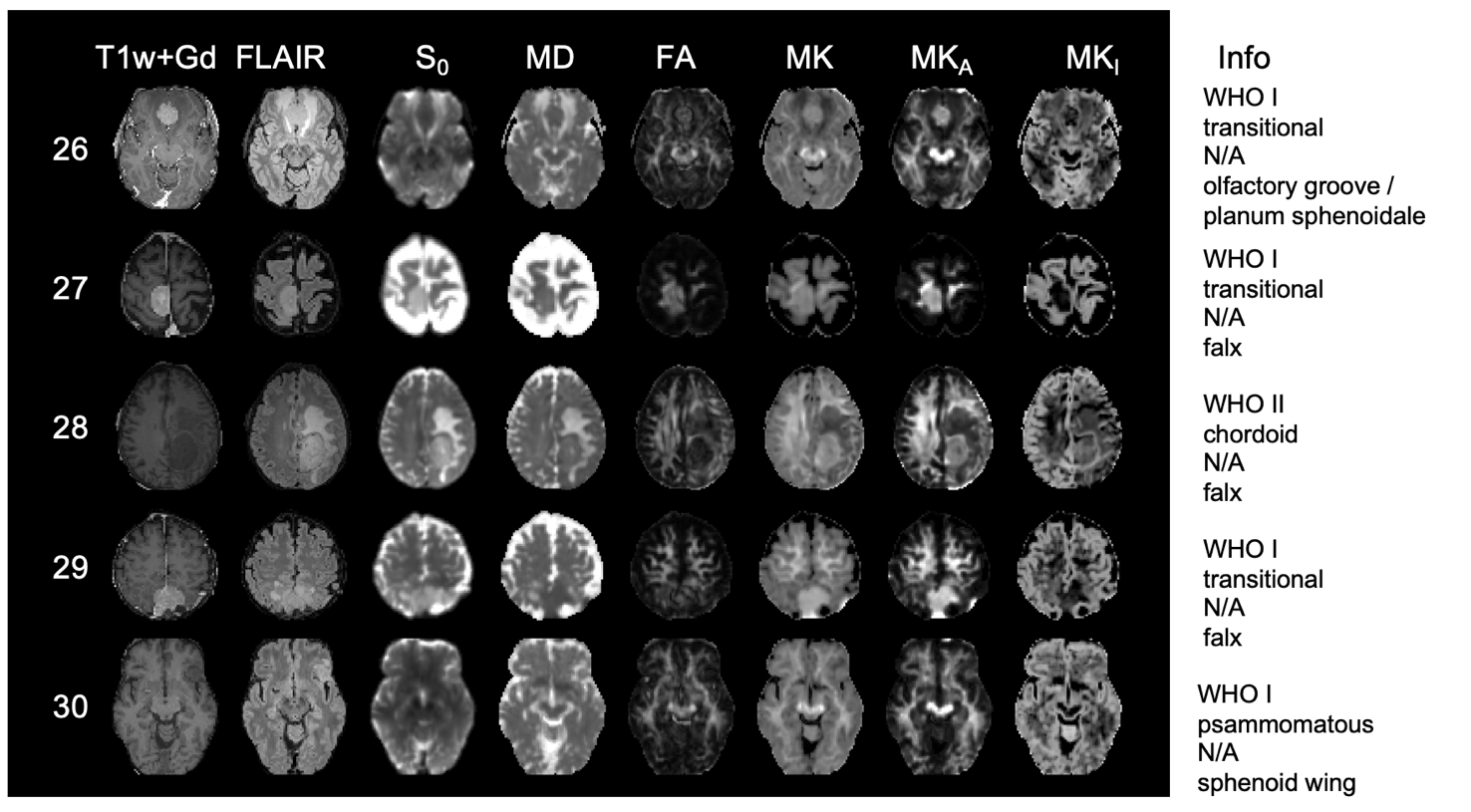


Figure 13. Complete overview of all the cases, part 6. Case 28 and 30 lack post-Gd T1w scan. In the rightmost column information on the grade, type, consistency and location is shown.

# MK_I_-rim for grade prediction


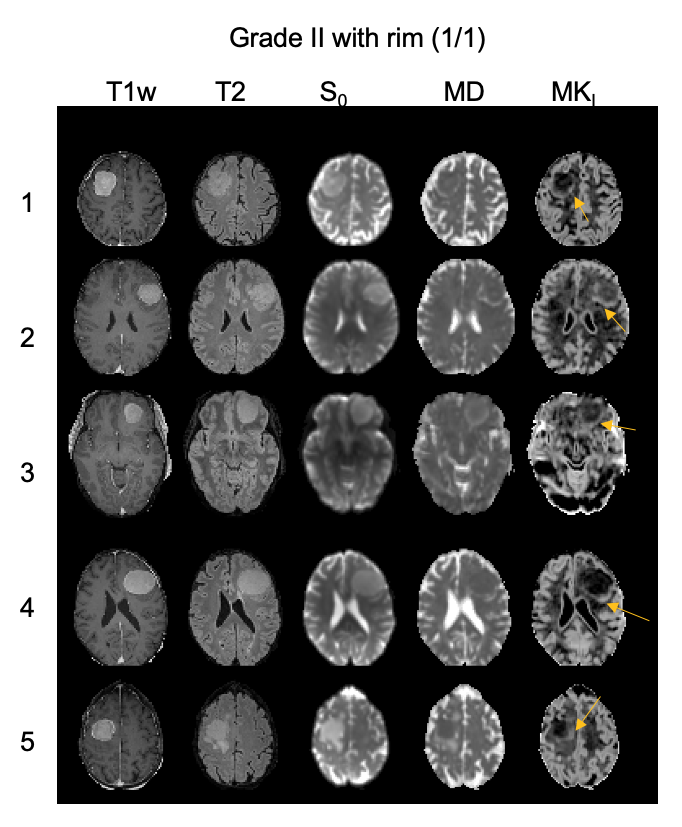


Figure 1. All cases of grade II that had a MK_I_-rim present around the tumor in the MK_I_ map are shown. The yellow arrow points at the location of the tumor rim.


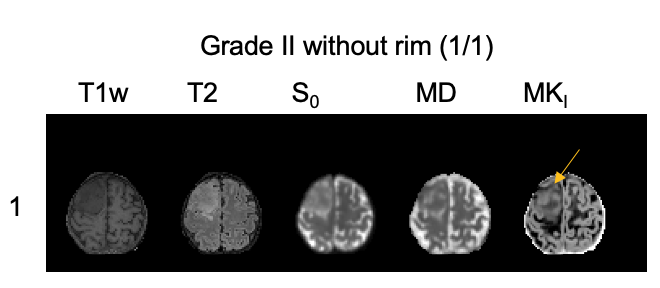


Figure 2. A case of grade II that did not have a MK_I_-rim present around the tumor in the MK_I_ map is shown. The yellow arrow points at the location of the tumor rim.

#
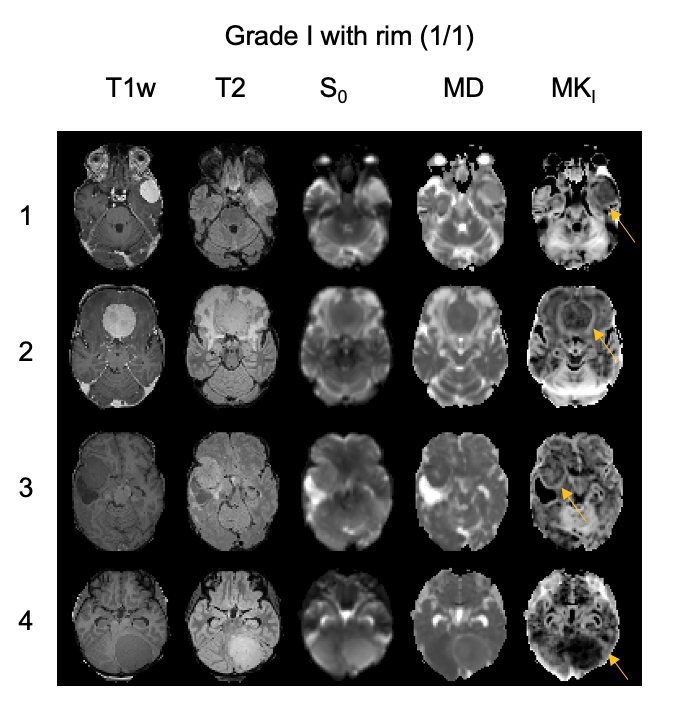


Figure 3. Cases of grade I with MK_I_-rim in the MK_I_ map are shown. The yellow arrow points at the location of the tumor rim.


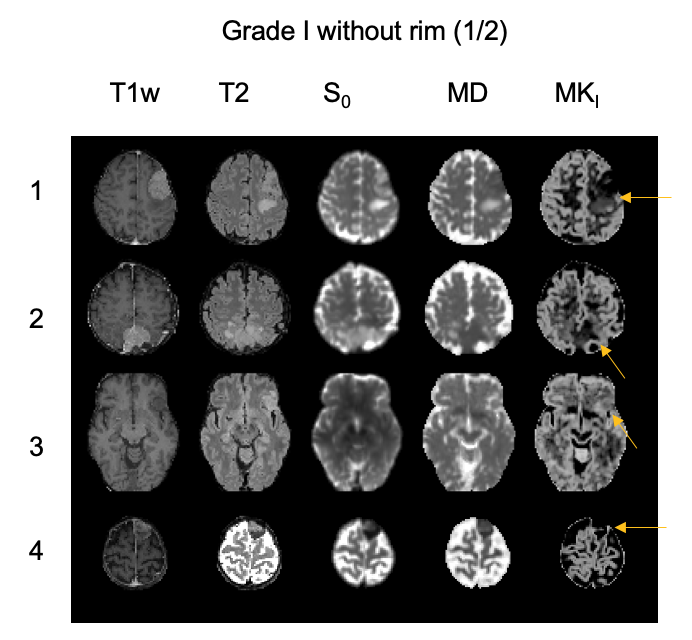


Figure 4. Cases of grade I without a MK_I_-rim in the MK_I_ map. The yellow arrow points at the location of the tumor region.


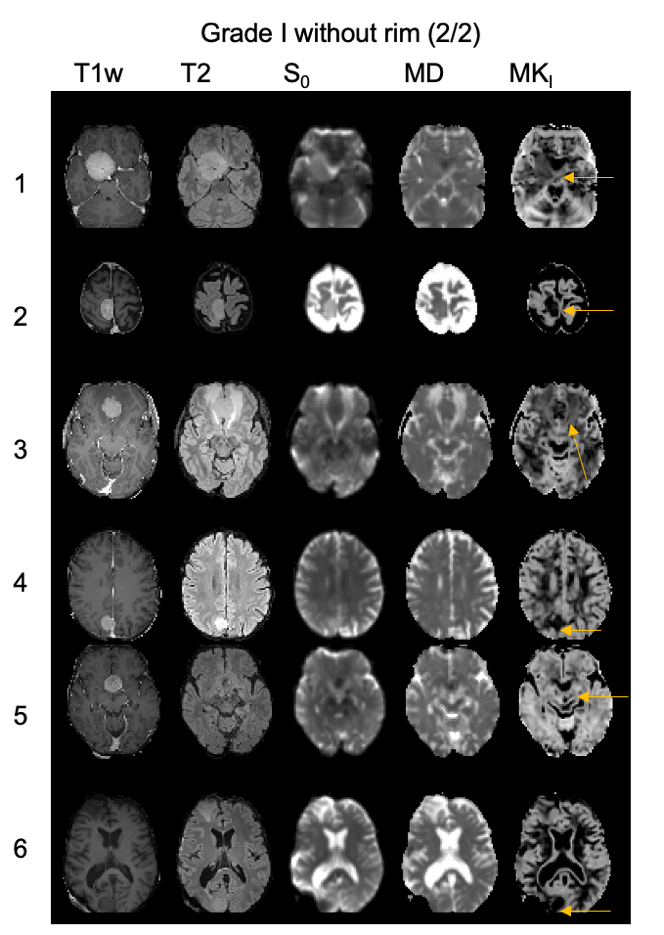


Figure 5. Cases of grade I without MK_I_-rim in the MK_I_ map are shown. The yellow arrow points at the location of the tumor region.


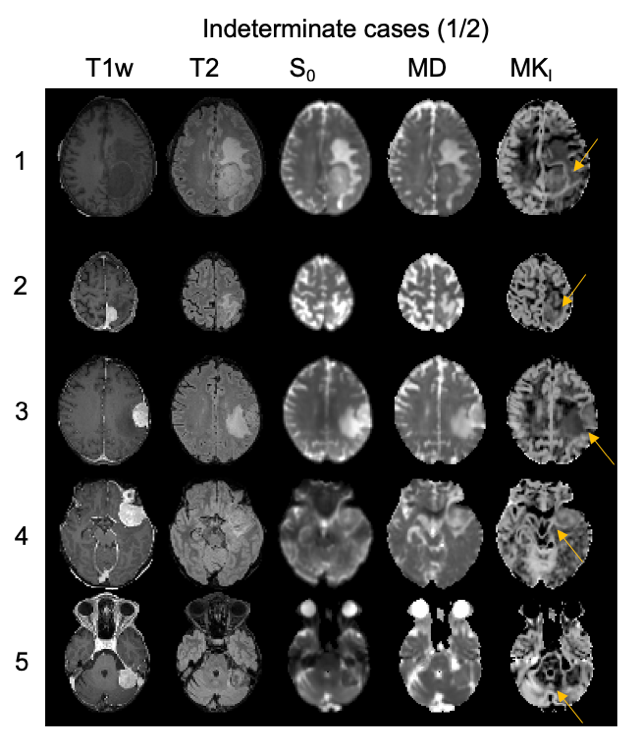


Figure 6. Interdeterminate cases that could be categorized with/without the presence of MK_I_-rim are shown. The yellow arrow points at the location of the tumor region.


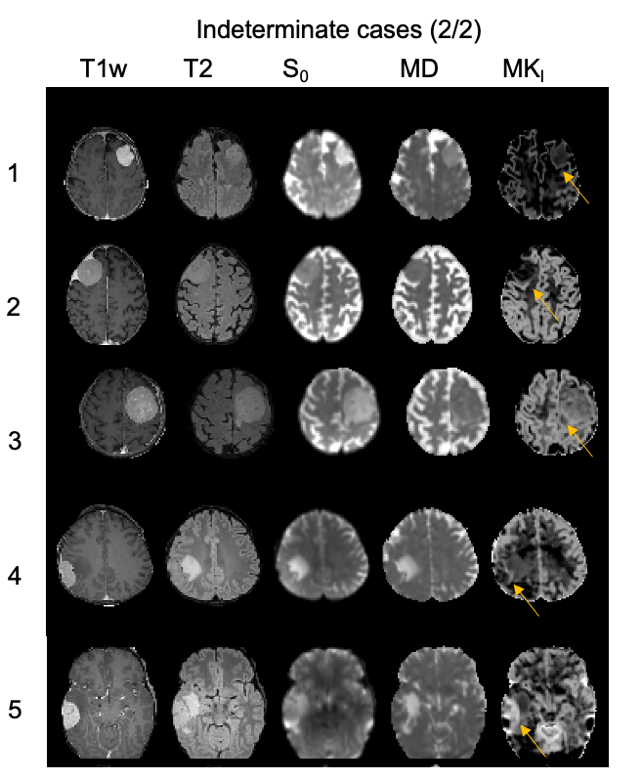


Figure 7. Interdeterminate cases that could be categorized with/without the presence of MK_I_-rim are shown. The yellow arrow points at the location of the tumor region.

# Statistics tables

| Distribution characteristics in  whole-tumor ROI | Significant parameters | | | Mean Cohen’s d |
| --- | --- | --- | --- | --- |
|  | Soft (n = 4) | Variable (n = 5) | Stiff (n = 7) |  |
| 10^th^ | - | MD | MK, MK_A_ | 0.56 |
| 25^th^ | - | MD | MK_A_ | 0.53 |
| 50^th^ (median) | - | MD | - | 0.50 |
| 75^th^ | - | MD | - | 0.47 |
| 90^th^ | - | MD | - | 0.50 |
| Standard deviation | FA | MK_A_ | - | 0.44 |

Table 1. Significant dMRI parameters in distinguishing meningioma consistency to the rest of the cases at significance level 0.05. U-Test. Univariate analysis.

| Distribution characteristics in  whole-tumor ROI | Significant parameters | | | | | | | Mean Cohens’ d |
| --- | --- | --- | --- | --- | --- | --- | --- | --- |
|  | Fibroblastic (1; n = 8) | Meningothelial (2; n = 3) | Transitional (3; n = 9) | Clear-cell (4; n = 2) | Microcystic/Angiomatous (5; n = 2) | Chordoid (6; n =1) | Psammomatous (7; n = 5) |  |
| 10^th^ | - | - | - | - | S_0_, MD, MK, FA, MK_A_ | - | - | 0.65 |
| 25^th^ | - | - | - | - | S_0_, MD, MK, FA, MK_A_ | - | - | 0.68 |
| 50^th^ (median) | - | - | - | - | S_0_, MD, MK, FA, MK_A_ | - | MK_A_, MK_I_, | 0.71 |
| 75^th^ | - | - | - | - | S_0_, MD, MK, FA, MK_A_ | - | MK_A_ | 0.69 |
| 90^th^ | - | - | - | - | S_0_, MD, MK, FA, MK_A_ | - | MK_A_ | 0.64 |
| Standard deviation | MK | FA | - | - | FA | - | S_0_ | 0.51 |

Table 2. Significant dMRI parameters in distinguishing meningioma type to the rest of the cases at significance level 0.05. U-Test. Univariate analysis.

Table 3. Significant dMRI parameters in distinguishing grade I from grade II at significance level 0.05. U-Test. Univariate analysis.

| Distribution characteristics  in rim-ROI | Significant parameters | Mean Cohens’ d |
| --- | --- | --- |
|  | Grade I (n = 22) vs grade II (n = 8) |  |
| 10^th^ | - | 0.44 |
| 25^th^ | - | 0.40 |
| 50^th^ (median) | - | 0.33 |
| 75^th^ | - | 0.29 |
| 90^th^ | - | 0.23 |
| Standard deviation | MK_I_ (p = 0.04) | 0.36 |

# Consistency and meningioma type

Table 4. Association between meningioma type and consistency. Table shows that the consistency is spread among most of the meningioma types.

| Type/  Consistency | Fibroblastic | Meningothelial | Transitional | Clear-cell | Microcystic/Angiomatous | Chordoid | Psammomatous |
| --- | --- | --- | --- | --- | --- | --- | --- |
| Number of cases | 8 | 3 | 9 | 2 | 2 | 1 | 5 |
| soft | 2 | 1 | 1 | 0 | 0 | 0 | 0 |
| variable | 2 | 1 | 1 | 0 | 0 | 0 | 1 |
| stiff | 1 | 0 | 1 | 2 | 2 | 0 | 1 |


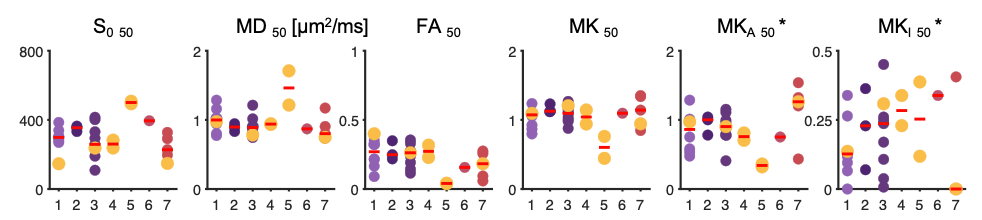


Figure 14. Association between meningioma type and consistency. Figure shows median distribution. The yellow dots show meningioma with stiff consistency (7 cases) which distributed throughout the different types. 1 = Fibroblastic, 2= Meningothelial, 3 = Transitional, 4 = Clear-cell, 5 = Microcystic/Angiomatous, 6 = Chordoid, 7 = Psammomatous.

# Grade association in the convexity meningiomas

Table 5. Significant dMRI parameters in distinguishing grade I from grade II at significance level 0.05 in the convexity only. U-Test. Univariate analysis. The only significant parameter is MK of the 25^th^ percentile.

| Distribution characteristics  in rim-ROI | Significant parameters | Mean Cohens’ d |
| --- | --- | --- |
|  | Grade I (n = 6) vs grade II (n = 5) |  |
| 10^th^ | - | 0.83 |
| 25^th^ | MK (p = 0.03) | 0.87 |
| 50^th^ (median) | - | 0.89 |
| 75^th^ | - | 0.87 |
| 90^th^ | - | 0.79 |
| Standard deviation | - | 0.36 |

# Parameter tables

Table 6. Overview of parameter values with respect to meningioma consistency. *Variable.


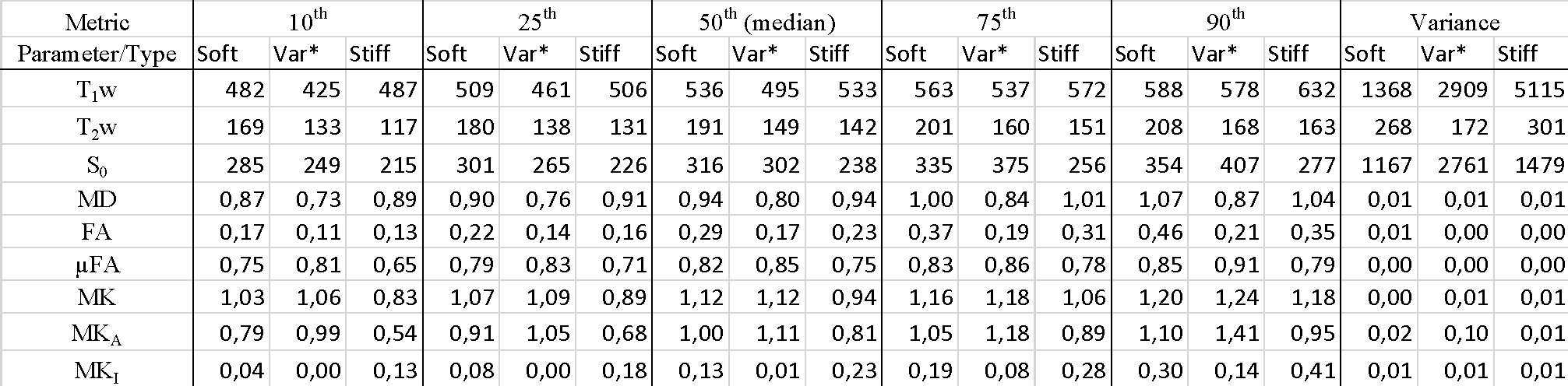


Table 7. Overview of parameter values with respect to meningioma type. 1* = Fibroblastic, 2* = Meningothelial, 3* = Transitional, 4* = Clear-cell, 5* = Microcystic/Angiomatous, 6* = Chordoid, 7* = Psammomatous.


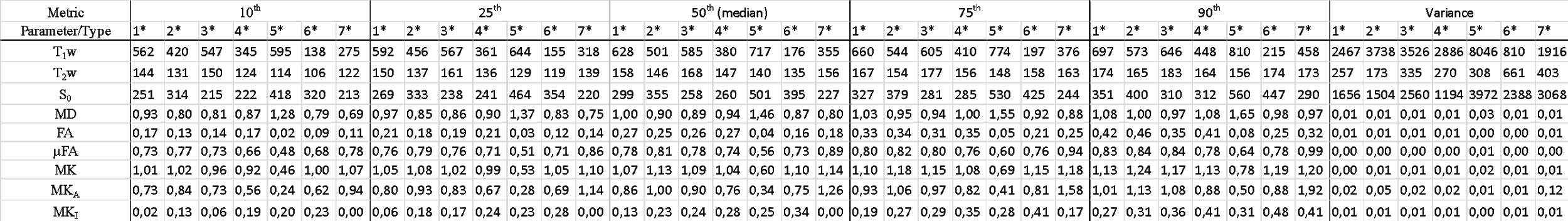


Table 8. Overview of parameter values with respect to meningioma grade.


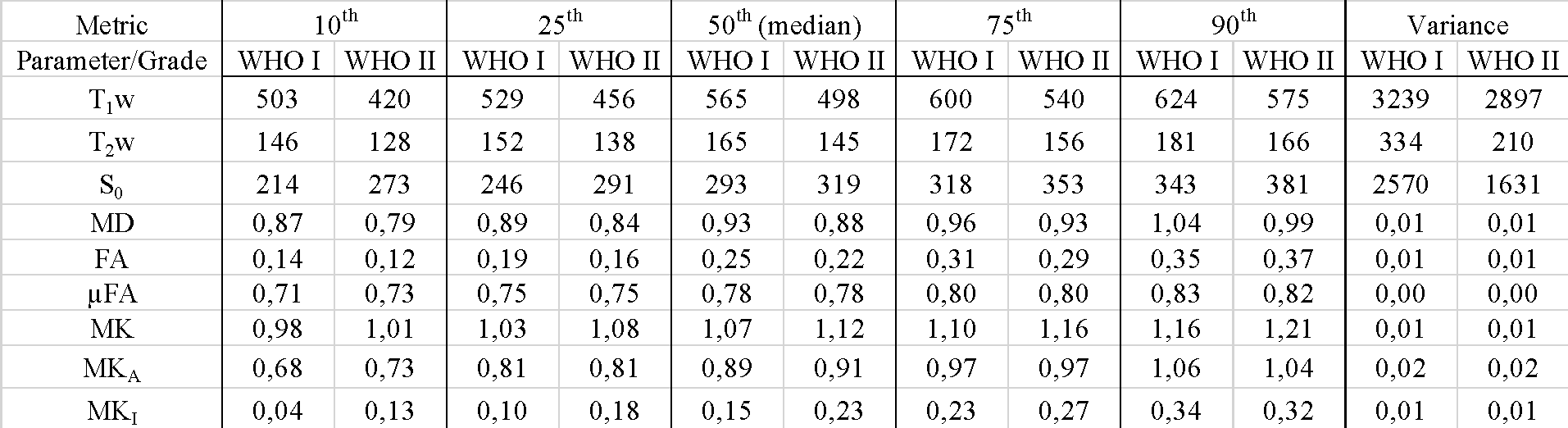

Supplement: Supplementary Data 1 [file mmc1.docx]
